# Enzyme-Catalyzed Oxidation of 17**β**-Estradiol Using Immobilized Laccase from *Trametes versicolor*

**DOI:** 10.4061/2011/725172

**Published:** 2011-08-22

**Authors:** Chantale Cardinal-Watkins, Jim A. Nicell

**Affiliations:** Department of Civil Engineering and Applied Mechanics, McGill University, 817 Sherbrooke Street West, Montreal, QC, Canada H3A 2K6

## Abstract

Many natural and synthetic estrogens are amenable to oxidation through the catalytic action of oxidative enzymes such as the fungal laccase *Trametes versicolor*. This study focused on characterizing the conversion of estradiol (E_2_) using laccase that had been immobilized by covalent bonding onto silica beads contained in a bench-scale continuous-flow packed bed reactor. Conversion of E_2_ accomplished in the reactor declined when the temperature of the system was changed from room temperature to just above freezing at pH 5 as a result of a reduced rate of reaction rather than inactivation of the enzyme. Similarly, conversion increased when the system was brought to warmer temperatures. E_2_ conversion increased when the pH of the influent to the immobilized laccase reactor was changed from pH 7 to pH 5, but longer-term experiments showed that the enzyme is more stable at pH 7. Results also showed that the immobilized laccase maintained its activity when treating a constant supply of aqueous E_2_ at a low mean residence time over a 12-hour period and when treating a constant supply of aqueous E_2_ at a high mean residence time over a period of 9 days.

## 1. Introduction

Endocrine disruption is a problem of increasing environmental importance, as evident by anomalies that have been discovered in wildlife exposed to a variety of exogenous toxic compounds released into the aquatic environment via municipal and industrial effluents and agricultural runoff [1, 2]. Estrogens excreted by humans and entering aquatic systems via sewage treatment plants are of particular interest since estrogen excretion cannot currently be controlled at the source, and estrogens are among the most potent known endocrine disruptors [3]. 

Particularly problematic estrogens include the natural estrogens 17*β*-estradiol (E_2_), estriol (E_3_) and estrone (E_1_) and also the synthetic estrogen 17*α*-ethinylestradiol (EE_2_), which is the active ingredient in birth control pills and other therapeutic reagents. Such compounds are frequently found in wastewaters and in surface waters in quantities that are in excess of their predicted no-effect concentrations [4] and are reportedly responsible for the majority of the estrogenic activity of municipal wastewaters [5, 6]. Given that these estrogens are phenolic compounds, they are amenable to oxidation through the catalytic action of a variety of oxidative enzymes [4]. Thus, it has been postulated that there is potential for applying enzymes to treat problematic estrogens commonly found in waste streams [4, 7–9]. 

The recognition of this potential has recently prompted researchers to explore the technical feasibility of this process. Much of this work was directed toward evaluating the ability of plant peroxidases and fungal laccases to treat aqueous-phase estrogens in bench-scale batch reactors (e.g., [4, 10]). While peroxidase enzymes have demonstrated relatively high redox potentials and can achieve appreciable conversion of their target substrates, even at very low concentrations in the nanomolar to micromolar range [4, 9, 11], their catalytic activity requires the use of hydrogen peroxide as an oxidant. In contrast, laccase enzymes have a lower redox potential, but have the advantage of using readily-available molecular oxygen as an oxidant [12]. Also, substrate conversion can be enhanced when laccase is used in conjunction with various noncatalytic cooxidants known as mediators [13]. Of the many laccases that may be selected as candidate enzymes for treatment processes, laccase from *Trametes versicolor* exhibits amongst the highest redox potentials [14] and has demonstrated a very good ability to remove substrates such as phenol, bisphenol A, and the estrogens E_2_, EE_2_, and E_3_ from aqueous solutions [10, 12, 15].

The use of enzymes for wastewater treatment has many potential advantages including the substrate specificity of enzymes, no susceptibility of the biocatalyst to shock-loading effects (i.e., since it is a biochemical system rather than a biological system), high rates of reaction, efficient use of oxidants, and effective treatment at low substrate concentrations [16, 17]. While the use of enzymes for converting waste compounds has many advantages, there are also some drawbacks that could limit their application, such as the gradual inactivation of the enzyme over time, inactivation of the enzyme due to side reactions in reacting mixtures, and interference of wastewater contaminants with enzymatic reactions [18]. Moreover, when the aqueous enzyme is used to treat contaminants in waste streams, the high cost of continually discarding the enzyme with treated solutions may be particularly prohibitive. 

In earlier work with peroxidase and laccase, these enzymes were found to be quite robust in terms of their ability to catalyze the oxidation of substrates in the presence of many wastewater contaminants and under wide ranges of pH and temperature [15, 18, 19]. Inactivation was found to occur with both types of oxidase enzymes, but rates of inactivation were found to be low when substrate concentrations were low [4, 20, 21]. These findings are quite promising in terms of the treatment of estrogens that are present in wastewaters at very low concentrations. However, the losses associated with the release of the soluble enzymes in the treated effluents still represent a significant limitation that must be addressed in order to minimize catalyst costs. Thus, it is hypothesized that immobilizing the enzymes onto a solid support for continual use in a reactor system could mitigate some of these problems. Immobilization is a process whereby enzyme is attached or adsorbed onto a water-insoluble matrix, thereby, retaining the enzyme in a reactor and exposing it to a continuous flow of substrate over time.

In order to attempt to minimize the amount of enzyme required to achieve effective treatment of estrogens, the objective of the present study was to characterize the conversion of E_2_ using *T. versicolor* laccase immobilized onto controlled porosity silica beads and used in a small-scale flow-through reactor. E_2_ was chosen as a model substrate because it is the most potent of the natural female hormones, and previous studies found very promising trends in the conversion of this and other estrogens [4, 10, 22]. The impacts of the following system conditions on the conversion of 17*β*-estradiol (E_2_) by laccase immobilized in a packed bed reactor were investigated: pH, contact time, initial E_2_ concentration, and temperature. The stability of the enzyme over time and under varying conditions of pH and temperature was also studied.

All experiments were conducted using influent E_2_ concentrations much higher than that which are typical of wastewater treatment plant influents; that is, experiments were conducted in the mg/L range, whereas E_2_ has been found in treatment plant effluents in the ng/L range [23]. This was done in order to establish clear and informative trends, while working within the confines of available analytical equipment. However, the influent E_2_ concentrations used in experiments are quite close to those found in human female urine and so the results may be relevant if treatment were to be performed at the source [24].

## 2. Materials and Methods

### 2.1. Chemicals and Reagents

Laccase from *T. versicolor* (EC 1.10.3.2) was purchased from Fluka (Ronkonkoma, NY). The nominal activity quoted by the manufacturer was 0.72 U/mg, where 1 unit (U) corresponds to the amount of enzyme which converts 1 *μ*mol of catechol per minute at pH 6.0 and 25°C. The dry enzyme powder was stored in a sealed container in a refrigerator at 4°C until needed. 17*β*-estradiol (E_2_) (>98% purity) was purchased as a dry powder from Sigma-Aldrich (St. Louis, MO). Concentrated stock solutions of E_2_ were prepared in methanol and stored in a refrigerator at 4°C until needed. Glutaraldehyde solution (25%, Grade II), glycine, and American Chemical Society (ACS) grade phosphoric acid (85% in water) were purchased from Sigma-Aldrich (St. Louis, MO). Sodium chloride and boric acid powder were purchased from Fluka (Ronkonkona, NY). Sodium hydroxide (1 N) and sulphuric acid (1 N) were purchased from Fisher (Fair Lawn, NJ). Glacial acetic acid (HPLC grade) was purchased from Caledon Laboratories (Georgetown, ON). Britton-Robinson buffer was used to maintain the desired pHs of all reaction solutions. The buffer consisted of a solution of 0.1 M acetic acid, 0.1 M boric acid, and 0.1 M phosphoric acid, which was then adjusted to the desired pH by addition of 1 N sodium hydroxide. All reagents and buffers were prepared using distilled-deionized water produced using a D4741 Nanopure Water System from Barnstead/Thermolyne (Dubuque, IA).

Methylene blue powder purchased from Anachemia (St-Laurent, QC) was used in all tracer studies. A concentrated methylene blue solution was prepared in distilled water and stored until needed. More dilute solutions of 4.8 mg/L concentration were prepared in distilled water on the day of the tracer study.

CPC- (controlled porosity carrier-) silica carrier silane-coated beads were purchased from Fluka (Ronkonkona, NY). As indicated by the manufacturer, these beads were derivatized with 3-aminopropyl-triethoxysilane and were reported as 30 to 45 mesh with a pore size of 375 Å. Since the beads are sensitive to humidity, they were stored in sealed containers in a cool, dry place until use.

### 2.2. Immobilization

Laccase was immobilized on the CPC-silica beads using a method reported previously [25]. First, 8 g of beads were immersed in 2.5% glutaraldehyde in a stoppered filtration flask and put under light vacuum for 2 hours in order to degas the beads and allow as much bead surface area as possible to be coated with aldehyde groups. Subsequently, the beads were thoroughly washed with pH 5 buffer (prepared as described above) and then immersed in a stock laccase solution (i.e., 200 mg of laccase in 200 mL of buffer prepared the same day) for at least 18 hours at 4°C. After thoroughly washing the beads with buffer again, the beads were rinsed with 0.5 M sodium chloride in order to prevent sorption of the enzyme onto the beads, since only the measurement of reactions catalyzed by covalently bonded enzyme was desired. The beads were again thoroughly washed with buffer and then immersed in 2.5 mg/mL glycine for at least 18 hours at 4°C. Once again, they were thoroughly washed, first with buffer, then 0.5 M sodium chloride, then buffer, and finally stored in buffer at 4°C until use. All reagents used in the immobilization process were made in pH 5 buffer.

### 2.3. Chemical Analyses

Tracer studies were conducted in order to determine the mean-residence time of the immobilized-enzyme reactor system and the time it took for the reactor effluent to reach a steady-state concentration. During tracer studies, the absorbance of the methylene blue in the effluent was measured using a Hewlett Packard 8453 diode array spectrophotometer that measured absorbance at a wavelength of 624 nm. The influent and effluent methylene blue concentrations (i.e., with a maximum concentration of 6.4 mg/L) always fell within the linear portion of the calibration curve (*R* ^2^ = 0.999).

The concentration of E_2_ in samples was determined using a High Performance Liquid Chromatograph (Agilent HPLC 1100 Series) fitted with a reverse-phase column (Zorbax SB-C18 Column). An injection volume of 20 *μ*L was used. A carrier phase consisting of 60% HPLC-grade acetonitrile and 40% distilled-deionized water was maintained at a flow rate of 0.8 mL/min. This isocratic flow was maintained at 39°C and was analyzed by the fluorescence detector (FLD) for 8 minutes. The FLD was set at an excitation wavelength of 230 nm and an emission wavelength of 310 nm.

### 2.4. Experimental Protocols

#### 2.4.1. Reactor Setup

The immobilized enzyme reactor system consisted of Kontes Chromaflex 2.5-cm inner diameter, 15-cm length columns, fitted with various accessory fittings, flow adapters, and 1/8 inch inner diameter polytetrafluoroethylene (PTFE) tubing purchased from Fisher (Fair Lawn, NJ). Tygon 3/32 inch inner diameter tubing was also purchased from Fisher (Pittsburgh, PA). Additional fittings to join the two types of tubing were purchased from Mandel (Guelph, ON). When only low flows or small total flow volumes were required, the flow was injected at a constant rate using a syringe pump (Harvard Apparatus 22 models) fitted with 50-mL glass syringe. When a continuous flow over an extended period was required, the flow was pumped using a Waters Millipore Model 510 pump from a stirred and covered reservoir and into a flow adapter and through the packed bed. 

The beads onto which laccase had been immobilized were placed into the bottom of a column. A flow adapter was inserted into the column and placed above the beads so that the beads were immersed in buffer and also such that a layer of 2-3 mm of buffer and 2-3 mm of air headspace existed above the bed. This resulted in a reactor media volume of 14.7 cm^3^, and liquid and air headspace volumes of 1.0–1.5 cm^3^ each. An 85-cm length of PTFE tubing was attached to the inlet of the reactor. When a syringe pump was being used to inject flow into the reactor, the end of the PTFE tubing was further attached by a fitting to 10 cm of Tygon tubing which could easily be attached to a 50-mL syringe.

#### 2.4.2. Reactor Characterization

Before any experiments with E_2_ were undertaken, studies were conducted to characterize the reactor using silica beads (i.e., in their original state without immobilized enzyme) set up in a reactor as described above. 

The porosity (*n*) of the packed bed of silane-coated silica beads was determined according to the water saturation method. Three trials gave an average porosity of 0.68 ± 0.03. 

Tracer studies were performed at each of the flow rates at which reaction experiments were performed in order to characterize the flow through the reactor and to determine the time needed for the reactor to reach equilibrium under each flow condition. For each of the different flow rates, water was passed through the column and, at time zero, a constant flow of water at the desired flow rate containing 6.4 mg/L methylene blue was introduced. Samples of effluent were taken intermittently and analysed using by spectrophotometry. The mean residence time, *t*_res_, and variance, *σ* ^2^, of the reactor under each flow condition were evaluated using a method described previously [26]. These characteristics, along with each empty bed contact time (EBCT) and the ideal contact time assuming plug flow conditions (CT), are presented in Table 1. As can be seen in Table 1, the variance increased with the mean residence time, indicating that the flow characteristics deviated from plug flow conditions as the mean residence time increased.

Also measured was the time to reach 95% of the equilibrium (i.e., the effluent tracer concentration reached 95% of the influent concentration) at each flow rate, as shown in Table 1. The time to reach 95% of equilibrium (*t*_95%_) was used as a basis for sampling in subsequent experiments because in reactor systems with low flow rates, and hence high mean residence times, the time to reach full (100%) equilibrium was inordinately long. In experiments in which time was the independent variable, this was not a concern, as the experiments were conducted for much longer than the time required to achieve 95% of equilibrium. Otherwise, in experiments where samples were taken after the time to 95% equilibrium, triplicate samples, with time lags between each sampling time, were taken and analyzed for E_2_ content.

#### 2.4.3. Reaction Experiments

For reaction experiments, an influent stock solution with the desired concentration of E_2_ and pH was prepared and continuously stirred in a partially covered container during an entire experiment. No significant losses of E_2_ from the container were observed over the course of the experiments. Once the pump was started, sampling of the reactor effluent commenced only after the system had reached near equilibrium, as had been pre-determined through the tracer studies described above. For all samples, 1 mL of effluent was collected and immediately acidified with 100 *μ*L of sulphuric acid to reduce the pH to below 2.5. This acidification step immediately halted any catalytic reaction that may be caused if some of the immobilized laccase had leached into the effluent thereby catalyzing further oxidation of E_2_ before sample analysis.

## 3. Results

In order to explore the use of immobilized laccase for the oxidation of E_2_ in aqueous solutions, experiments were conducted to determine the effects of pH, temperature, mean residence time, and initial substrate concentration on E_2_ conversion. Studies were also conducted to study the short-term and long-term stability of immobilized laccase under various working conditions.

In order to compare the efficacy of the enzyme at achieving E_2_ conversion under various conditions such as pH and temperature and over time, it was necessary to ensure that the same quantity of enzyme was available for each experiment. At present, it is not possible to ensure that the quantity of enzyme immobilized on a given quantity of solid media in a reactor column is the same between experiments. Thus, it was not possible to directly compare results achieved using different columns. Therefore, in experiments conducted to measure the effects of selected variables, all experiments were run using the same column. However, when studying the effect of a control variable such as pH on substrate conversion, it was necessary to ensure that the enzyme activity in the reactor did not decrease during or between successive experiments. At present, no enzyme activity assay is available to directly monitor the activity of immobilized laccase. However, it is possible to measure the activity indirectly by passing a solution with fixed pH, temperature, initial substrate concentration and flow rate through the column and measuring the conversion of that substrate achieved under these conditions. If the quantity of substrate converted by the reactor declines over time, this indicates that the activity in the column is also declining. This indirect measurement of activity will be used throughout experiments discussed below. 

### 3.1. Effect of pH on Substrate Conversion

In order to be effective as a catalyst in applications involving the conversion of a target substrate, laccase must not only be stable under various environmental conditions such as pH, it must also be able to exert its catalytic action on a target substrate. Therefore, studies were conducted to optimize the conversion of E_2_ over a range of pH. However, given earlier findings that the optimal conditions for laccase stability as well as conversion of phenolic substrates in the aqueous phase are under slightly acidic to neutral conditions [10, 12, 15], the effect of pH on immobilized laccase was studied over a limited pH range of 4 to 7.

**Figure 1:**
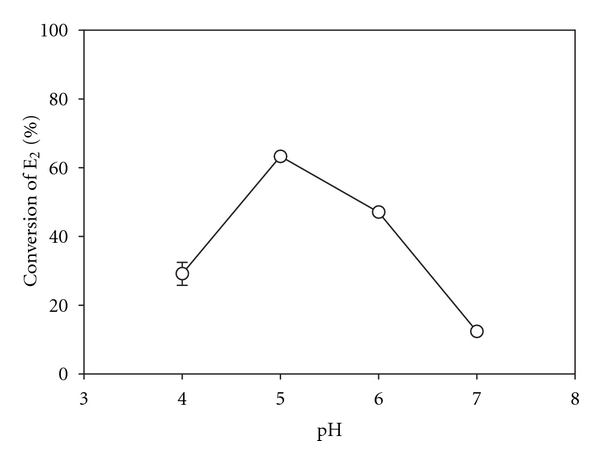
Effect of pH on the conversion of E_2_ in the packed bed reactor. Reaction conditions: 4 g dry media, 19.2 U/g nominal activity, [*E*_2_]_*i*_ = 10.0 ± 0.5 *μ*M (influent estradiol concentration), 21°C, EBCT = 4.2 minutes. All points represent the mean of three samples taken at 2-minute intervals during the same experiment with error bars representing one standard deviation from the mean.

An experiment was conducted in which 10-*μ*M solutions of E_2_ were prepared in buffers ranging from pH 4 to 7 and passed through the reactor in order to assess conversion of E_2_. The initial concentration of the stock solution was measured using HPLC. Between experimental runs at each pH, a solution of E_2_ in pH 5 buffer was passed through the reactor and a sample of the effluent E_2_ concentration was measured in order to ensure that enzyme activity had remained relatively constant over the course of the experiment. Thus, the pH experiments were conducted in the following order: pH 5, pH 7, pH 5, pH 6, pH 5, and pH 4. Each part of the flow sequence was run for 40 minutes, and at each pH, a series of three samples were taken, each two minutes apart. The effect of pH on the conversion of E_2_ by immobilized laccase is shown in Figure 1.

The effect of pH on the conversion of E_2_ by immobilized laccase follows the same trend as was observed previously for laccase in the aqueous phase [10]; that is, conversion was optimal at pH 5, followed closely by high conversion at pH 6. Though the optimum of pH 5 does not fall into the typical range of pHs for domestic wastewaters, which is from pH 6.7 to pH 7.5 [27], this pH was used for most subsequent experiments in order to optimize reaction conditions for the use of laccase. However, some experiments that involved the measurement of enzyme stability over time were performed at pH 7, as it has been found that, although conversion rates tend to be reduced at pH 7, the enzyme was observed to be more stable in the aqueous phase over a longer period at this pH [12].

**Figure 2:**
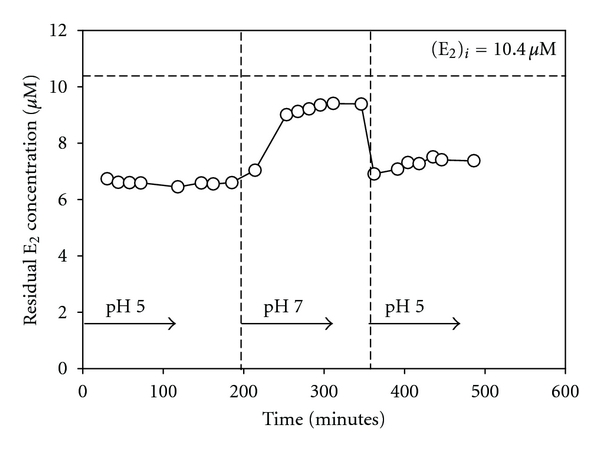
Effect of changing pH on the conversion of E_2_. Reaction conditions: 8 g dry media, 18.0 U/g nominal activity, *t*_res_ = 9.8 minutes, [*E*_2_]_*i*_ = 10.4 *μ*M, 21°C.

A subsequent experiment was run in which approximately 10 *μ*M E_2_ in pH 5 buffer was passed through the reactor, followed by a solution of the same concentration at pH 7, and then again at pH 5. For each period with flows at different pH levels, the effluent E_2_ concentration was measured over time. The purpose of this experiment was to assess the impact of varying the pH in the reactor on the activity of the enzyme. The results are shown in Figure 2. Several trials of the experiment were run, and all showed a similar trend. The results of this experiment demonstrate that much more conversion occurs at pH 5 than at pH 7, which is consistent with the results reported above in Figure 1. They also showed that, although step changes in pH of the solution through the reactor did not have a large impact on conversion achieved by the enzyme, conversion declined somewhat after the pH was brought back to pH 5 from pH 7. In this experiment, the conversion of E_2_ achieved after equilibrium was established at pH 5 was approximately 35%, whereas after the system was exposed to pH 7 and then returned to pH 5, the conversion declined to 28%. The results demonstrate that the step changes to which the enzyme was exposed caused a moderate decrease in activity.

### 3.2. Reaction Kinetics

**Figure 3:**
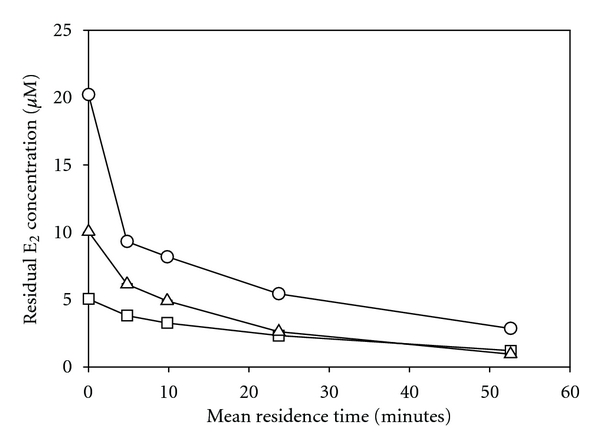
Conversion of E_2_ as a function of mean residence time for three influent concentrations. Reaction conditions: 8 g dry media, 18.0 U/g nominal activity, at 21°C and pH 5 with initial estrogen concentrations, [E_2_]_i_, of (□) 5 *μ*M, (∆) 10 *μ*M, and (○) 20 *μ*M. All points represent the mean of three samples taken at 5-minute intervals during the same experiment with error bars representing one standard deviation from the mean (not visible due to their small size).

**Figure 4:**
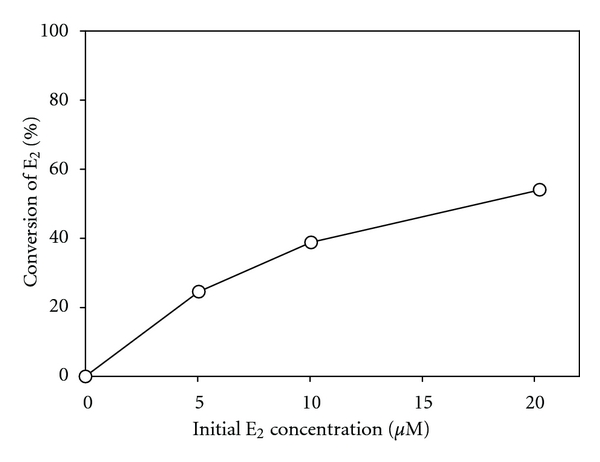
Conversion of E_2_ as a function of initial E_2_ concentration at a mean residence time of 4.8 minutes. Reaction conditions: 8 g dry media, 18.0 U/g nominal activity, 21°C and pH 5. All points represent the mean of three samples taken at 5-minute intervals during the same experiment with error bars represent one standard deviation from the mean (not visible due to their small size).

Three separate experiments using different influent concentrations of E_2_ (i.e., 5, 10, and 20 *μ*M) were conducted in which substrate conversion was assessed as a function of mean residence time. The results are shown in Figure 3. As expected, as the mean residence time increased, the amount of E_2_ converted also increased. Only a short residence time of approximately five minutes was required to achieve moderate conversion of E_2_ (e.g., greater than 50% in the case where [*E*_2_]_*i*_ = 20 *μ*M). This residence time was used in subsequent experiments because a moderate level of conversion in the control was deemed desirable when evaluating the impacts of process variables on treatment effectiveness; that is, under this fixed condition, it is possible to observe both positive and negative impacts of other variables (e.g., pH, temperature, time) on treatment. Figure 4 presents the relative conversion of E_2_ at a mean residence time of 4.8 minutes for each tested value of initial substrate concentration. From this graph, it is evident that the relative conversion of E_2_ increases as its initial concentration increases.

### 3.3. Temperature

The effect of temperature is an important variable to be examined in laccase-catalyzed reactions because it can have a twofold effect. Firstly, most enzymes are inactivated at elevated or even moderate conditions of temperature and some can be deactivated at temperatures at or below 10°C [12, 28]. Thermal inactivation is believed to be mainly caused by denaturing of the tertiary structure of the enzyme either through protein unfolding or disruption of the active site of the enzyme [28, 29]. Secondly, according to the Arrhenius Law, the rate of reaction should tend to increase with temperature. Therefore, there are two simultaneous effects associated with changes in temperature in a reacting system: (1) a change in the rate of reaction over time caused by thermal inactivation of the enzyme and (2) a change in the reaction rate due to Arrhenius effects. 

**Figure 5:**
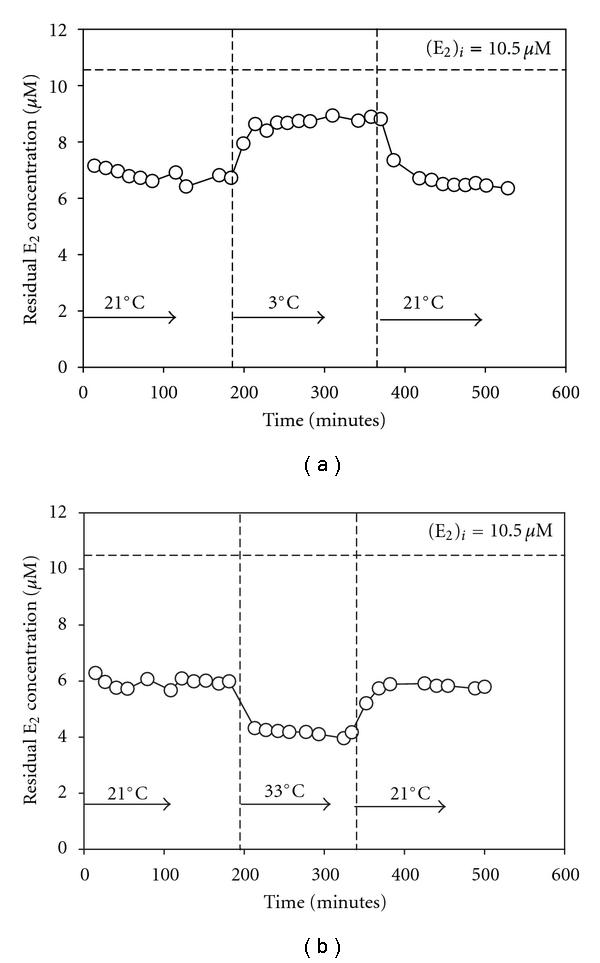
Effect of changing temperature on E_2_ conversion accomplished by immobilized enzyme for temperature changes (a) from ambient (21°C) to cold (3°C) and back to ambient, and (b) from ambient to warm (33°C) and back to ambient. Reaction conditions: 8 g, 18.0 U/g nominal activity, pH 5.

Therefore, experiments were conducted to characterize the ability of immobilized enzyme to convert E_2_ at different temperatures, as well as to assess the impact of step changes in temperature on the enzyme's stability. The ambient temperature of the reactor was 21°C. In one experiment, the reactor was operated at the ambient temperature for several hours after which a step change in the temperature of the flowing fluid to 3°C was made and the conversion of E_2_ was then monitored over time for approximately 3 hours. Thereafter, the system was returned to the ambient temperature and the system was again monitored for an additional period of several hours. This experiment was then repeated but with an intervening step-change of reaction temperature to 33°C. In both cases, the step change in temperature involved quickly transferring the reactor to a room set at the desired temperature and supplying the reactor with the same E_2_ stock solution that had previously been equilibrated at that temperature. The results of these experiments are shown in Figure 5. 

As can be seen in Figure 5, the time for the enzyme reactor to respond to step changes in temperature was quite brief. In both cases, whether reverting from 3°C back to 21°C or reverting from 33°C back to 21°C, the enzyme appeared to return to a level of activity similar to that measured before the step change in temperature. Figure 5(a) shows that reducing the temperature from 21°C to 3°C reduced the activity of the immobilized enzyme by approximately 20%, whereas Figure 5(b) shows that increasing the temperature from 21°C to 33°C increased its activity by approximately 20%. Given that the enzyme was quite stable under these temperature conditions, it is evident that within the range of temperatures observed, Arrhenius effects dominate in the reacting system.

### 3.4. Enzyme Stability

The use of enzymes immobilized on a support for oxidation of substrates is being studied primarily for its potential as a means for reducing the total amount of enzyme that is required to treat a continuous flow of contaminated water. As such, an important criterion for reducing the amount of enzyme required to accomplish treatment is whether the enzymes immobilized on a support remain active for a substantial period of time. To satisfy this criterion, it is essential that (1) the enzyme be stable when stored on the support for extended periods of time prior to or between uses and (2) the enzyme be stable under reaction conditions. Thus, these two aspects of laccase stability were studied, as shown below.

#### 3.4.1. Stability under Storage Conditions

The results shown above demonstrate that the pH optimum for laccase-catalyzed conversion of E_2_ is at pH 5, but it is also expected that treatment of E_2_ would often be required/preferred at pH 7 given that this is more typical of many wastewaters [27]. In prior studies in which soluble laccase (i.e., nonimmobilized) was used to oxidize phenol in aqueous solutions, it was found that although the enzyme's catalytic activity was higher at pH 5 than at pH 7, its stability was much greater at pH 7 [12]. Thus, an experiment was conducted using two separate reactors where one was always subjected to pH 5 solutions and the other always subjected to pH 7 solutions. Each reactor was used periodically for 3-hour runs over a 3-month period and was stored under conditions of no flow at 25°C, with one reactor stored with its media in pH 5 buffer and the other with its media in pH 7 buffer. 

**Figure 6:**
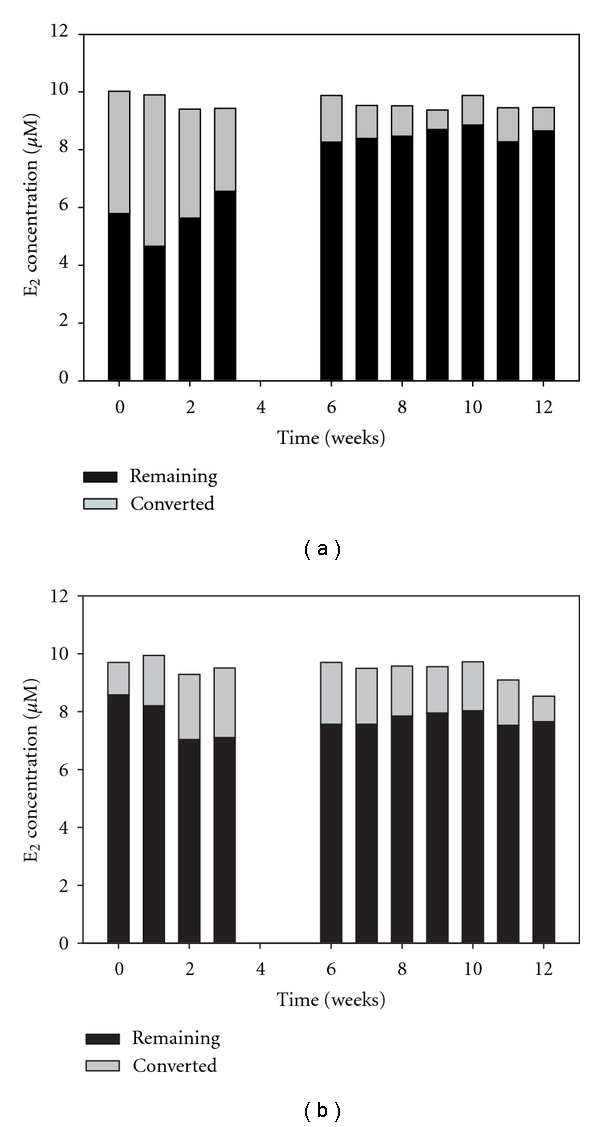
Remaining and converted E_2_ after periodic 3-hour runs of the substrate through an immobilized-enzyme packed bed reactor stored at 25°C and (a) pH 5 or (b) pH 7 for extended periods of time. Reaction conditions: 8 g dry media, 18.0 U/g nominal.

The results of this experiment are shown in Figure 6. As expected, higher conversion was initially achieved in the reactor runs conducted at pH 5 due to the greater activity of the enzyme toward the target substrate under this condition. However, a much larger decline in activity over time is seen in the reactor runs at pH 5, shown in Figure 6(a), than in the reactor runs at pH 7, shown in Figure 6(b).

#### 3.4.2. Inactivation of Immobilized Laccase under Reacting Conditions

An experiment was conducted to assess the stability of the immobilized enzyme over a 12-hour period of continuous flow of substrate through the reactor. The results shown in Figure 6 indicated that the enzyme was more stable (i.e., retained its activity better) under storage conditions at pH 7 than at pH 5. While it is more likely to be practical to run an immobilized enzyme treatment system over the long term at pH 7, it might also be desirable to operate the system at pH 5 for moderate lengths of time. Two experiments were therefore conducted to assess the stability of laccase under these conditions. 

**Figure 7:**
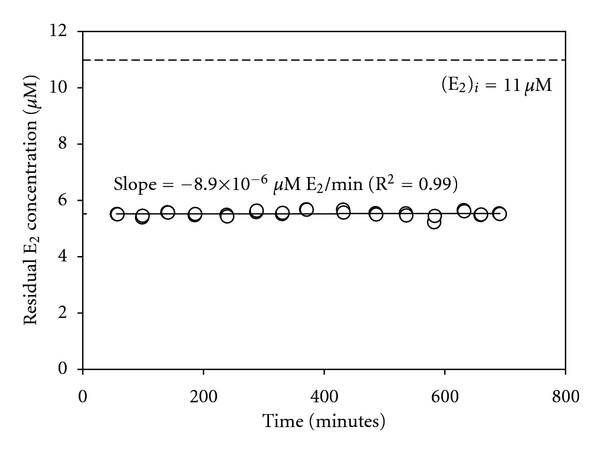
Stability of immobilized enzyme over a 12-hour period at pH 5 with continuous flow of E_2_. Reaction conditions: 8 g dry media, 18.0 U/g nominal activity, *t*_res_ = 24 minutes, [*E*_2_]_*i*_ = 11.0 *μ*M at 21°C.

Firstly, the reactor was run for a period of approximately 12 hours at pH 5. The results are shown in Figure 7. A linear regression fit of the data gives a slope of −8.9 × 10^−6^ *μ*M E_2_/minute, with a regression coefficient (R^2^) of 0.999. The very low slope (i.e., a measure of the rate of change of conversion of E_2_ by the reactor over time), which is essential zero given the precision of E_2_ measurements and other sources of experimental error, indicates that insignificant inactivation of the enzyme occurred over this period of operation at pH 5. 

Secondly, because the results from Figure 6 suggest that it might be more feasible to operate a reactor of immobilized laccase over the long term at pH 7, a final experiment was undertaken to assess a reactor's ability to accomplish the oxidation of E_2_ with a continuous flow of influent at pH 7 over a longer period of 9 days. A long mean residence time of approximately 650 minutes was chosen for this study. This high residence time was chosen in order to determine whether a high degree of conversion of the target substrate could be achieved and maintained at pH 7, which is not optimal for the enzyme's catalytic activity, as was demonstrated above. 

**Figure 8:**
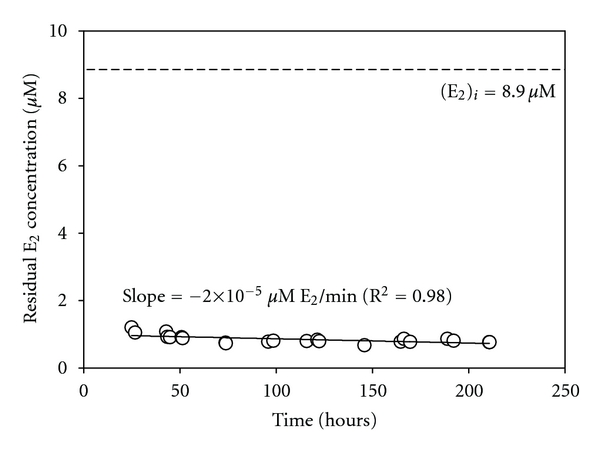
Stability of immobilized enzyme over a 9-day period at pH 7 with continuous flow of E_2_. Reaction conditions: 8 g dry media, 18.0 U/g nominal activity, *t*_res_ = 650 minutes, [*E*_2_]_*i*_ = 1.86 *μ*M at 25°C.

The results of this experiment, which are shown in Figure 8, demonstrate that the significant increase in mean residence time dramatically increased the conversion of E_2_ achieved by the reactor at pH 7. Moreover, the results show that under the specified conditions, the immobilized enzyme in the reactor was very stable over a 9-day period. A linear regression fit of the data produces a slope of −2.0 × 10^−5^ *μ*M E_2_/minute with an R^2^ of 0.988. Given the precision of the measurements and sources of experimental error, the slope is so close to zero as to be insignificant.

## 4. Discussion

The tracer studies for all flow rates used in this study were presented in Table 1. In all cases, the mean residence time of the nonreactive dye was longer than the ideal contact time (CT) for an ideal plug-flow reactor, with the greatest deviation occurring for the lowest flow rate, as seen by the ratios expressed in Table 1. These results suggest that there are many dead zones within the reactor, and that the volume of these dead zones and time spent by reacting solutions within them increases as the flow rate decreases. The occurrence of dead zones as well as channelling effects greatly impacts the quality of the reactor effluent, given that much of the enzyme in dead zones was not being used to its fullest capacity. If the reactor were to be optimized with respect to flow conditions, it is anticipated that the treated effluent could be of much greater quality. Thus, it is concluded that the data presented in this study is conservative and the conversion of E_2_ at any stated conditions could be greater if the reactor were fully optimized. 

There exist many ways to optimize a packed bed reactor so as to minimize dead zones. For example, the inlet structure could be redesigned such that flow would be more evenly distributed as it is introduced into the top of the packed bed. Alternatively, the geometry and the capacity of the reactor could be changed. A reactor with a higher flow rate will tend to decrease the effect of dead zones. A wider and longer column could also minimize wall effects. Therefore, for a desired residence time, the reactor could be optimized by increasing the flow rate and proportionately increasing the depth and/or width of the packed bed to achieve the desired residence time. Dead zones could also be reduced by employing a medium with different size and shape. 

The denaturation and consequent decrease in activity of enzymes at extremes of pH occurs because of folding of the tertiary structure that has ionisable side-chains [28]. In an earlier study, the optimal pH for the aqueous-phase laccase-catalyzed oxidation of phenol was found to occur at pH 6, with comparable oxidation also occurring at pH 5 [12]. In the case of bisphenol-A, the optimum pH was at 5 [15]. Similarly, the oxidation of E_2_ and ethynylestradiol (EE_2_) by laccase in the aqueous phase has been found to occur optimally at pH 5 [10]. A third estrogen, estriol (E_3_), follows a pattern closer to that of phenol [10]. In all cases, the conversion of the target substrates was optimal under slightly acidic conditions with reasonable conversion being achieved relative to the optimum in the pH range of 4 to 7. 

Similar results to those quoted above were found in this study: that is, the conversion of E_2_ occurred optimally at a pH of 5, followed closely by good conversion at a pH of 6. From this, it is evident that optimal performance by laccase would not be achieved within the typical range of pH for domestic wastewaters, which is from pH 6.7 to pH 7.5 [27]. Thus, in order to maximize performance, the pH of the influent solutions would either need to be decreased or other treatment parameters would need to be more adjusted to compensate for the lower activity of the enzyme under near-neutral conditions. The latter of these two options would seem more feasible and, based on results obtained pertaining to enzyme stability, preferable, since it was found that the enzyme exerts significant catalytic ability under neutral conditions (see Figure 8) and the enzyme system stored over the long term with only short intermittent uses is much more stable at pH 7 than at pH 5 (see Figure 6).

The greater stability of laccase at pH 7 has been noted elsewhere. For example, it was reported that aqueous laccase at pH 5 was moderately stable with an activity loss of approximately 10% after being stored for 6 hours, while at pH 7 it was very stable with an activity loss of approximately 3% in the same period [12]. In another study, it was also observed that the stability of stored laccase was highest at pH 6 and 7 [15]. Under the storage conditions of the present study, which lasted three months, a much greater discrepancy between stability at pH 5 and 7 was observed. In practical applications, where immobilized enzyme would most probably be stored for extended periods, perhaps more on the order of days or weeks than hours, storage at pH 7 would therefore be preferred to storage at pH 5.

Moderate recovery times (i.e., 5 minutes to 1 hour) were reported for aqueous-phase laccase exposed to extremes of pH and then brought back to neutral pH [12]. This has been thought to occur because of tangles formed during denaturation, which render the refolding of the tertiary structure more difficult [28]. As seen in Figure 2, there appears to be a moderate recovery period in the case of immobilized laccase as well. After the expected time required to achieve equilibrium elapsed (see Table 1), the conversion of E_2_ continues to show a sloping trend, possibly arising from a gradual return to its original conformation, when the pH is brought from 5 to 7 and then returned to pH 5. 

In addition to pH, the effects of another important influent parameter, temperature, on the reactivity and stability of laccase were studied. It was found that within the range of temperature from 3 to 33°C, immobilized laccase activity is positively correlated with temperature (see Figure 5). In the case of conversion of bisphenol-A by the same species of laccase in the aqueous phase, it was found that activity of the enzyme was positively correlated with temperature in the range of 25 to 45°C, and above 45°C the two became negatively correlated [15]. This suggests that over a very wide range of temperatures, Arrhenius' Law, stating that reactions increase with increasing temperatures, has a greater impact than the temperature-induced unfolding of the enzyme's tertiary structure. Only when a very high temperature is reached or exceeded (e.g., 45°C), does the negative impact of temperature become apparent. 

While, in general, the positive correlation with temperature exists for the reactivity of the enzyme, the opposite effect is observed when measuring the effect of temperature on enzyme stability. For example, dramatic decreases in storage stability were reported with increasing temperature [12, 15]. Also, it was determined that temperature-induced inactivation was greater in a reacting system than in a stored system [15]. While the stability of the enzyme based solely on temperature was not assessed in the present study, the practical implications of such results are very important because the relationship is very pronounced. For example, due to seasonal variations, most influent wastewaters in the United States fall within the range of 3 to 27°C [30]. Thus, although reactivity would be slightly higher at higher temperatures, the stability of the enzyme would be much greater during the tertiary treatment of typical domestic wastewaters. 

Although the stability of the immobilized enzyme system with respect to temperature was not studied over the long term, the recovery time for step changes in temperature was indirectly investigated. For example, it was reported that the recovery times when the nonreacting enzyme undergoes a step change in temperature were relatively long, ranging from 3 to 12 hours [12]. Similar to the case of pH, this recovery time would be due to the gradual untangling of side chains in the tertiary structure of the enzyme [28]. In this study, however, as seen in Figure 5, there was no clear evidence of a recovery time period. When step changes in temperature were introduced to an operating reactor, the enzyme appeared to achieve a steady activity after the expected time to steady-state as determined by tracer studies and no later. This could be due to a stabilizing effect of the enzyme in a chemically immobilized state. Alternatively, the data in the present study may not have been sufficiently precise to reliably observe a small change (i.e., <5%) in activity over time. 

The importance of stability during storage was pointed out above, but even more important in practical applications is operational stability. As seen in Figure 7, operating a reactor of immobilized laccase at the optimal pH (in terms of reactivity) of 5 did not result in any inactivation under the employed conditions. Since treatment is more likely to be conducted at or near neutral pH and over longer periods of time, the results in Figure 8 are more representative of what is more likely to occur in practice. At pH 7 and over 9 days, albeit with a high mean residence time of approximately 650 minutes (EBCT = 74 minutes), no enzyme inactivation was observed when continuously treating 10 *μ*M of E_2_.

A major limitation to using an immobilized enzyme system for tertiary treatment of domestic wastewaters was illustrated in Figure 4. Under the studied conditions, the lower the influent substrate concentration, the lower the conversion within the reactor. This likely arises from lower rates of reaction and slower rates of mass transfer at low substrate concentrations. Fortunately, significant treatment was accomplished in the immobilized-enzyme packed reactor with reasonable retention times. However, the influent concentrations used in these experiments were between 1 and 10 mg/L, whereas wastewater treatment plant effluents have been found to have E_2_ concentrations between 1 and 24 ng/L [23], which are six orders of magnitude lower. Thus, some other operational parameters would need to be greatly enhanced to compensate for the expected reduced reaction rates with lower estrogen concentrations; that is, the quantity of enzyme immobilized per unit volume of reactor would need to be significantly increased. In addition, the rate of mass transfer from the bulk solution to the immobilized enzyme would be much slower for environmentally relevant substrate concentrations. Thus, enhanced mixing and a dense distribution of the enzyme over greater surface areas would be required to offset these mass transfer limitations. These represent very significant challenges that must be overcome before the tertiary treatment of estrogens (or other phenolic contaminants at trace concentrations) using immobilized laccase can be considered practical. 

As an alternative, source treatment could be considered as an option, though this would represent a very significant change in waste management paradigm for urban communities. Such a paradigm shift is already being considered and promoted due to a variety of advantages and opportunities associated with the source separation, collection, and treatment of urine [24]. Human urine has an average E_2_ concentration of around 3 *μ*g/L [31], or three orders of magnitude lower than the experimental influent concentrations. Therefore, source treatment might improve the reaction kinetics dramatically over what would be possible with tertiary treatment, but mass transfer limitations may still prove to be extremely restrictive. 

Despite the limitations noted above for the treatment of estrogens, the results of this work are encouraging since it has been demonstrated that immobilized laccase is very stable when used to treat low concentrations of substrates. Therefore, this technology could also be considered for the treatment of a variety of problematic phenolic pollutants in various wastewaters.

## 5. Conclusions

The objective of this research was to address the growing problem of estrogens and other endocrine disruptors which pass unchanged through wastewater treatment plants and make their way into natural aquatic environments. Previous work has focused on treating these compounds using laccase in the aqueous phase. The main objective here was to explore enzymatic treatment a step further by immobilizing the enzyme onto a support such that it could be used retained in a continuous flow reactor instead of being discarded with treated effluents. The estrogen estradiol, E_2_, was used as a model substrate. 

The conversion of E_2_ was found to occur optimally at pH 5, whereas long-term storage stability was much greater at pH 7 than at pH 5. Because wastewaters are typically at or near neutral pH, real applications of the system could be used under the conditions which confer greatest stability. For the immobilized laccase system, E_2_ conversion was positively correlated with temperature in the range from 3 to 33°C, and for step changes in temperature, the system did not show any perceptible recovery period typical of an enzyme slowly regaining its conformation.

Kinetic experiments revealed that the contact time required for significant E_2_ conversion was relatively low, even in a nonoptimal reactor configuration, which is promising in terms of larger-scale operations. However, when treatment was conducted using a variety of influent concentrations of E_2_, it was observed that the rates of reaction slowed significantly with lower substrate concentrations. Given that the E_2_ concentrations used in these experiments were 3 to 6 orders of magnitude greater than what might be found in a real treatment applications, this is a significant problem which needs to be addressed before the feasibility of this approach can be established. It should also be noted that the reactor used in all experiments exhibited fairly poor flow characteristics. Therefore, improving the reactor design could lead to a significant increase in substrate conversion under all studied conditions.

The monitoring of the operational stability of the enzyme yielded promising results. In particular, a reactor run continuously at typical treatment conditions of pH 7 and at 25°C, albeit with high influent E_2_ concentration and relatively long contact times (i.e., 10 *μ*M, with an EBCT of 73.5 minutes and mean residence time of 650 minutes), resulted in high substrate conversion with no apparent inactivation over a 9-day period. To further assess the feasibility of the treatment process, the length of time that the reactor can be used before the immobilized enzyme needs to be replaced should be assessed. Studies are also needed to determine the magnitude of the impact of other contaminants in wastewater matrices on enzyme stability. 

The results of this study demonstrate that an immobilized reactor using laccase as a biocatalyst has potential to be used in the treatment of E_2_ and possibly many other phenolic substrates.

## Figures and Tables

**Table 1:** Summary of characteristics of the packed bed reactor system with a porosity (*n*) of 0.68 as determined using tracer studies at each of the influent flow rates used in all immobilized-enzyme experiments. Characteristics include empty bed contact time (EBCT), ideal contact time assuming plug flow conditions (CT), mean residence time (*t*_res_) and variance (*σ* ^2^), and time to 95% equilibrium (*t*_95%_). Also included is a measure of the disparity between mean residence time and ideal PFR contact time for different flow rates, as expressed by the ratio of *t*_res_/CT.

Influent flow rate (mL/min)	EBCT (min)	CT (min)	*t*_res_ (min)	*σ* ^2^ (min^2^)	Ratio *t*_res_/CT (dimensionless)	*t*_95%_ (min)
0.2	73.5	50.0	650	103 000	13.0	1400
1.0	14.7	10.0	53	730	5.2	160
2.0	7.3	5.0	24	110	4.7	65
4.0	3.7	2.5	9.8	28	3.9	19
8.0	1.8	1.2	4.8	8.3	3.9	11
